# Obesity and Systemic Inflammation Disrupt the Compensatory Role of Physical Activity in Chronic Pain Conditions

**DOI:** 10.3390/biomedicines13051111

**Published:** 2025-05-02

**Authors:** Taynah Galassi, Kevin Pacheco-Barrios, Anna C. Gianlorenco, Felipe Fregni

**Affiliations:** 1 Neuromodulation Center and Center for Clinical Research Learning, Spaulding Rehabilitation Hospital and Massachusetts General Hospital, Harvard Medical School, Boston, MA 02138, USA; taynahgalassi@hotmail.com (T.G.); kpachecobarrios@mgh.harvard.edu (K.P.-B.); gianlorenco@ufscar.br (A.C.G.); 2 Postgraduate Program in Health Sciences, Experimental Neuroscience Laboratory, University of Southern Santa Catarina, Palhoça 88137-272, Brazil; 3 Vicerrectorado de Investigación, Unidad de Investigación para la Generación y Síntesis de Evidencias em Salud, Universidad San Ignacio de Loyola, Lima 15024, Peru; 4 Postgraduate Program in Physical Therapy, Department of Physical Therapy, Federal University of Sao Carlos, São Carlos 13565-905, Brazil

**Keywords:** chronic pain, physical activity, sedentary behavior, body mass index (BMI), systemic inflammation, C-reactive protein (CRP), NHANES, pain prevention

## Abstract

**Objectives:** This study examines the influence of body mass index (BMI) and systemic inflammation on the relationship between physical activity and chronic pain conditions. **Methods:** We used data from the National Health and Nutrition Examination Surveys (NHANES, 2003–2004 and 2009–2010 waves), a population-based representative sample of the US population. Chronic pain conditions (neck, low back, hip, joint pain, and migraine) were defined as persistent pain for more than three months using self-reported questionnaires. Vigorous and moderate physical activity and sedentary time were collected using validated instruments. Weighted logistic regression models were used to test the adjusted associations and effect modifications. **Results:** We included 9809 individuals (mean age of 46.58 and 51% women). We found a protective adjusted association between vigorous physical activity and chronic neck, low back, and hip pain (2003–2004: OR = 0.798, 95% CI, 0.647–0.984; 2009–2010: OR = 0.629, 95% CI, 0.474–0.833). Consistently, higher sedentary time was associated with higher chronic pain prevalence. Likewise, vigorous physical activity was protective for chronic migraine pain (2003–2004: OR = 0.697, 95% CI, 0.517–0.939). However, it was not for chronic joint pain. Moderate physical activity does not have a protective association in our sample. Furthermore, this protective association was attenuated by high BMI levels (*p* = 0.011) and high CRP (*p* = 0.006). **Conclusions:** Vigorous physical activity has a protective association with chronic pain. People with obesity and high systemic inflammation presented an attenuated beneficial association. Our results suggest that pain condition, body composition, and systemic inflammation should be considered for the personalization of community-based physical activity interventions to prevent chronic pain conditions.

## 1. Introduction

Obesity and chronic pain are important public health priorities, with both prevalences growing annually in countries around the world [1,2]. Obesity is a chronic disease with a rising prevalence in both developed and developing nations. Excess body fat contributes to four million deaths annually and accounts for 120 million disability-adjusted life-years. Recent global estimates indicate that approximately 108 million children (~5% prevalence) and 604 million adults (~12% prevalence) are classified as obese [3]. Chronic pain is a debilitating condition that profoundly affects the lives of individuals and is associated with several comorbidities, including suicide and opioid-related mortality [4]. Nowadays, chronic pain affects over 100 million Americans—more than diabetes, cancer, and heart disease combined—with chronic musculoskeletal pain and migraine the most common forms of chronic pain [5,6].

Physical activity is highly recommended by healthcare professionals and healthcare systems for a wide range of conditions involving chronic pain [7,8]. There is evidence that physical activity is capable of reducing the severity of pain, improving physical function and also has positive effects on psychological function and quality of life [9]. On the other hand, these results are not found in all studies [10,11,12]. Reviews for this outcome conclude that the level of evidence is of low quality and inconsistent [13]. Therefore, there is a need for understanding of the sources of heterogeneity, particularly using population-based data.

Obesity may be one of the missing links between physical activity and pain. Obesity is defined by the World Health Organization as an abnormal or excessive fat accumulation that increases mortality risk and has been associated with chronic pain and multiple mental health complications [4]. The concurrent presence of obesity and chronic pain could exacerbate the patient’s functional status and quality of life to a greater extent than either condition alone [14]. Recent evidence indicates that the increasing prevalence of obesity contributes to the expanding disparity in chronic pain conditions [15], and other studies suggest that this higher chronic pain prevalence is contributing to maintaining higher levels of obesity and sedentary behaviors [16]. Those hypotheses are not necessarily mutually exclusive [17]; thus, the complex interplay between these two conditions represents a challenging clinical scenario.

Weight status and different degrees of inflammatory states are plausible factors influencing the effects of physical activity on chronic pain since there are part of postulated mechanistic pathways [18,19,20]. However, the role of high body mass index (BMI) and systemic inflammation as moderators in the relationship between physical activity and chronic pain is unclear. To the best of our knowledge, there is no population-based study assessing this relevant question, which could provide insights into personalizing physical activity interventions to prevent chronic pain at a populational level.

The research hypothesis of the present study is that physical activity is associated with a protective effect against chronic pain. However, obesity and inflammation may interfere with the protective effect of physical activity against the development of chronic pain. The purpose of this study is to examine the influence of BMI and systemic inflammation in the relationship between physical activity and chronic pain conditions in a large representative U.S. population, using the 2003–2004 and 2009–2010 cycles of the National Health and Nutrition Examination Survey (NHANES).

## 2. Materials and Methods

### 2.1. Data Source and Sample

The analytical data utilized in this study is publicly available and was obtained from National Health and Nutrition Examination Surveys (NHANES), specifically from the years 2003 to 2004 and 2009 to 2010. The period analyzed was chosen according to the availability of population data regarding moderate and vigorous physical activity. NHANES are a combination of cross-sectional population-based surveys, designed to assess the health and nutritional status of a representative sample of the civilian, non-institutionalized US population. The NHANES interviews and questionnaires include demographics, socioeconomic, dietary, and health-related questions. Medical and physiological measurements, as well as laboratory tests, are administered by trained medical personnel. NHANES examine a nationally representative sample of about 5000 persons each year, providing important information to meet emerging needs, determine the prevalence and risk factors of major diseases, be used in epidemiological studies and health sciences research, help to develop sound public health policy, as well as direct and design health programs and services.

**Figure 1:**
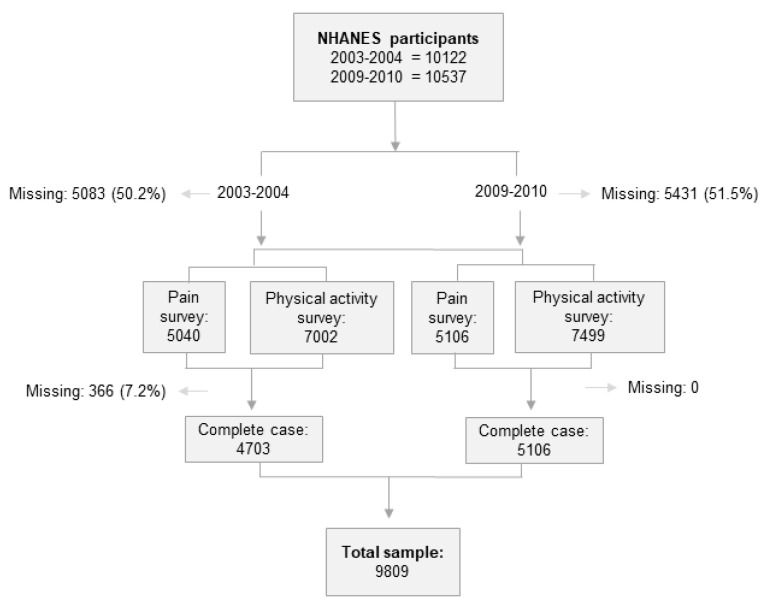
Flowchart of the research sample selection process.

From 2003 to 2004 a total of 4703 individuals and for 2009–2010, 5106 individuals participated in the NHANES, collected between January and December of each year. A total of 9809 individuals were included in the study. Age, gender, race/ethnicity, socioeconomic status, body measures, physical activity, as well as physical and emotional limitations, and dietary components are some of the parameters that can be extracted from the NHANES database for the study participants. The process of sample selection is presented in Figure 1.

### 2.2. Demographic Variables

Demographic characteristics included gender, age in months at screening, marital status (married, widowed, divorced, separated, never married or living with partner), race/ethnicity (Mexican American, other Hispanic, Non-Hispanic White, Non-Hispanic Black, other race- including multi-racial) and country of birth (US States or Washington/DC, Mexico, other Spanish-speaking country or other non-Spanish speaking country). The educational level was divided into five categories: less than 9th grade, 9–11th grade, high school or equivalent, some college or AA degree, college graduate or above. Self-reported annual family income was divided into four categories: $0 to $19.999, $20.000 to $24.999, $45.000 to 54.999, $75.000 and over, and also analyzed using the ratio of family income to poverty.

### 2.3. Health Status

For measuring the health status, we used the self-reported physical, mental and emotional limitations from the question “Are you limited in any way in any activity because of a physical, mental or emotional problem?”. For sleep disorders, “yes” was considered positive answer for “Have you ever been told by a doctor or other health professional that you have a sleep disorder?” Regarding alcohol use, consumption was based on the answer to the following question “In the past 12 months, how often did you drink any type of alcoholic beverage? How many days week, per month, or per year did you drink?”. Analysis of smokers was based on self-reported responses to the question “During the past 30 days, on the days that you smoked, about how many cigarettes did you smoke per day”.

### 2.4. Chronic Pain Outcomes

In this study, data for neck, low back, and hip chronic pain was collected from 2009 to 2010 NHANES and was defined by the affirmative answer to the question: “Have you ever had back, neck and/or hip pain almost every day for at least 6 weeks in a row?”. From 2003 to 2004, the data extracted regarding pain was related to joint pain and migraine and were defined by the affirmative answer to the questions: “During the past 12 months, have you had pain, aching, stiffness or swelling in or around a joint?” and “During the past 3 months, did you have severe headaches or migraines?”, respectively. Also, for neck and low back pain, was defined by an affirmative answer to the questions: “During the past 3 months, did you have neck pain?” and “During the past 3 months, did you have low back pain?”, respectively.

### 2.5. Physical Activity Exposure

In this study, vigorous physical activity was measured through the answer to the question “Does your work involve vigorous-intensity activity that causes large increases in breathing or heart rate, for at least 10 min continuously?”. Moderate physical activity was measured through the answer to the question “Does your work involve moderate-intensity activity that causes small increases in breathing or heart rate, for at least 10 min continuously?”, following the 2018 Physical Activity Guidelines Advisory Committee scientific report, which declares that participants who reported less than 10 min of moderate-to-vigorous physical activity per week should be classified as inactive [21,22]. Moreover, we included a self-reported measure of sedentary time on a typical day, including sitting at work, at home, getting to and from places, or with friends, including time spent sitting at a desk, traveling in a car or bus, reading, playing cards, watching television, or using a computer. The reported time was categorized in quartiles to improve interpretation.

### 2.6. Obesity and Systemic Inflammation

To quantify and analyze obesity, BMI was used, which is calculated during physical examination and in this study, divided into 2 groups (non-obese < 30 kg/m^2^, and obese ≥ 30 kg/m^2^) [23]. Waist circumference was used as predictor to visceral obesity (≥88 cm in women and ≥102 in men) [24]. Additionally, we included the serum C-reactive protein (CRP, mg/dL) measure from the blood sample assessments, as an indirect measure of unspecific systematic inflammation.

### 2.7. Dietary Inflammatory Index (DII)

The DII was calculated following the method described by Shivappa and colleagues [25], using dietary data collected for 24 h (midnight to midnight) on day 1, which were collected by MEC, and includes information on the type and amount of food and drinks consumed.

The DII was calculated considering 28 dietary components and aims to evaluate the level of systemic inflammation using six important inflammation markers (levels of serum IL-1β, IL-6, IL-4, IL-10, TNF-α and CRP). A score of “+1” was assigned if a dietary component increased the levels of pro-inflammatory cytokines and a score “−1” if a dietary component decreased the levels of pro-inflammatory cytokines or increased the levels of anti-inflammatory cytokines. Each individual food parameter was multiplied by its respective effect score derived from literature review [25]. The sum of all food parameter-specific scores resulted in the overall DII score for each participant and the pooled DII represents the pro-inflammatory or anti-inflammatory potential of an individual’s daily diet. For comparability of total energy intake, the E-DII was calculated for every 1000 calories of food consumed.

### 2.8. Data Analysis

For descriptive purposes, all available observations were included, following a complete-case analysis approach. All descriptive data were summarized with mean and standard deviation, with prior confirmation of the normal distribution of outcomes. Additionally, categorical variables were described in frequency and proportions.

In accordance with the proposed objectives of this study, we sought to develop an exploratory model. The following variables were identified as potential theoretical confounders for the relationship between physical activity and chronic pain prevalence: age, sex, alcohol, smoking, education level, SES, health status, and obesity. Direct acyclic graphs (DAGs) were sketched to explore the association of interest, and all potential confounders listed above. The study involved constructing DAGs to identify potential confounders, colliders, and mediators.

We constructed weighted logistic regression models using a stringent forward-selection process. Odds ratios were calculated with 95% confidence intervals. The level of statistically significant results was *p* < 0.05. First, unadjusted models were developed, including the physical activity measures (vigorous and moderate physical activity) as exposure and the chronic pain categories as outcome (neck, low back, hip pain, joint pain and migraine). Then, a multivariable model adjusted for all potential confounders was performed, unless covariate collinearity was detected.

Potential interactions were explored in the adjusted models. In particular, we tested the BMI level and CRP level categories by adding the interaction term in the models (BMI*physical activity or CRP*physical activity). If a significant interaction was identified, then stratified analysis were performed. In a sensitivity analysis, we tested similar models but including as exposure the quartiles of the sedentary time.

Statistics package R 2.11, with customized scripts were employed for complex survey analyses (survey and nhanesA packages).

## 3. Results

The sample size and baseline characteristics of the study participants of 2003–2004 and 2009–2010 NHANES are illustrated in Table 1. The mean age of study population was 48.9 years for 2003–2004 and 44.24 years for 2009–2010, with an almost equal distribution between the sexes, with 51.5% women and 48.4% men, and the majority being non-Hispanic white, born in US or Washington DC (78%).

Regarding lifestyle, the majority of the studied population exhibited a pro-inflammatory dietary pattern (52%), engaged in regular moderate physical activity (52% for 2003–2004 and 34% for 2009–2010), and had a BMI below 30 kg/m^2^ (67% for 2003–2004 and 61% for 2009–2010). The outcomes of interest for pain were neck/low back/hip pain, as well as migraine or severe headaches and joint pain, with a prevalence of 37.25% (2003–2004) and 29.53 (2009–2010), 19.96% and 45.16%, respectively.

### 3.1. Physical Activity Association with Neck, Low Back, and Hip Pain

Our unadjusted analysis for vigorous physical activity showed that physical activity has a protective effect against neck, low back, and hip pain reducing 25% (OR 0.74) of chances in developing pain for 2009–2010, and a similar result for 2003–2004, with a reduction of 18% (OR 0.82) in chances of developing neck, low back, and hip pain. In order to better measure this association, we performed linear regressions for neck, low back, and hip pain (dependent variable) and vigorous physical activity (independent variable), adjusted for the following covariates: gender, age, physical and emotional limitations and sleep disorders (Table 2). We found that physical, mental and emotional limitations (*p* = 0.010) and sleep disorders (*p* = 0.014) appeared to be independent predictors. Our adjusted analysis showed that physical activity has a higher protective effect, reducing 39% (OR 0.61) of chances in developing pain for 2009–2010, and 21% (OR 0.75) for 2003–2004.

Regarding moderate physical activity, our unadjusted analysis showed a significant reduction of 25% (OR 0.75) of chances in developing neck, low back, and hip pain, only for 2003–2004. After the adjustments, the result did not survive.

The association of moderate and vigorous physical activity for migraine and joint pain are illustrated in Table 3. Vigorous physical activity appears to have a protective effect reducing 22% (OR 0.78) chances of developing migraines, and after adjustments for gender, age, BMI, and DII, the protective effect is higher, showing a reducing of 31% (OR 0.69) chances of developing migraines. Gender, age, and DII are significant independent predictors. Our adjusted analysis showed that vigorous physical activity is a risk factor, increasing the chances of developing joint pain in 36% (OR 1.36).

### 3.2. BMI as an Effect Modifier

The role of BMI as an effect modifier was explored for the same outcomes (Table 4). Our regressions showed that BMI is an effect modifier in the relationship between physical activity and neck, low back, and hip when tested as a continuous (*p* = 0.04) variable, with even higher significance when tested as a categorical (*p* = 0.01) variable (BMI ≥ 30 kg/m^2^). Our stratified-adjusted analysis for 2009–2010 (Table 4) showed that the protective effect of physical activity is only seen in BMI < 30 kg/m^2^ (underweight, normal weight and overweight), reducing 47% (OR 0.57) of chances in developing neck, low back, and hip pain, and for obese subjects (BMI ≥ 30 kg/m^2^), the protective effect of physical activity is not statistically significant (*p* = 0.09, O.R. 0.75).

Similar results were found in our stratified-adjusted analysis for 2003–2010, where physical activity presented a protective effect of 37% (OR 0.63) against neck and low back pain for non-obese population (BMI < 30 kg/m^2^). For analysis considering BMI ≥ 30 kg/m^2^, the results were not statistically significant (*p* = 0.05, O.R. 0.92) and consistent with those found previously for obese population.

### 3.3. CRP Serum Levels as an Effect Modifier

CRP as an effect modifier for the protective effect of vigorous or moderate physical activity in neck, low back, joint pain, and migraine was explored. No significant statistical results were found in the relation between CRP levels, vigorous physical activity, and neck, low back, hip, and joint pain. Although, our regressions showed that CRP serum levels are an effect modifier in the relation between the protective effect of vigorous (*p* = 0.006) physical activity and chronic migraine (Table 5). Our stratified-adjusted analysis showed that the protective effect of physical activity is only seen when CRP serum levels are low, reducing 44% (OR 0.56) of chances in developing migraine.

### 3.4. DII Association with Pain

No significant association was observed between DII, the protective effect of vigorous or moderate physical activity and neck, low back, and hip pain in our adjusted analysis for age, gender, physical, mental and emotional limitations and sleep disorders, when tested for 2009–2010. The same is true for our adjusted analysis regarding the relation between DII, the protective effect of vigorous or moderate physical activity and joint pain or migraine.

### 3.5. Sensitivity Analysis

To test the robustness of our results, we explored similar associations but using the self-reported sedentary time variable as complement of the physical activity measures. Consistently, in the 2009–2010 cycle, we found that participants with higher quartile of sedentary time reported had 11% more odds of reporting neck, low back, and hip chronic pain, compared to participants from a lower quartile (OR = 1.11, 95% CI: 1.01–1.22, *p* = 0.03), suggesting a dose–response association between sedentary time and the prevalence of chronic pain.

## 4. Discussion

The main findings of this study support the protective effect of physical activity in chronic pain, as well as the role of BMI and CRP serum levels moderating this relationship. This research discovered that vigorous physical activity has a protective effect on neck, lower back, and hip pain (OR ranges from 0.629 to 0.798) in the US population. Importantly, this relationship is modified according to BMI level. BMI levels smaller than 30 make this relationship even stronger, while BMI levels equal or higher than 30 seem to block the association between physical activity and chronic pain. Similar effects were seen for migraine. Vigorous physical activity also presents a high protective association with chronic migraine (OR = 0.697), although it is a potential risk factor for the presence of joint pain (OR = 1.366). Our results were consistent with sensitivity analyses using sedentary time, where higher sedentary time quartiles were associated with more frequency of neck, low back, and hip pain (OR = 1.11). These findings align with the interpretation of odds ratios, where values below 1 indicate a protective association, as seen with vigorous physical activity and various types of pain, while values above 1—such as for joint pain and sedentary time—suggest an increased risk. Moderate physical activity does not have a protective association with chronic pain conditions in our sample.

We found that the protective association of physical activity with neck, low back, and hip pain is highly moderated by BMI levels, being present only in non-obese populations (BMI < 30 kg/m^2^). These results were found in both analysis for different waves of NHANES. Also, the protective role of vigorous physical activity against chronic migraine is only present when CRP serum levels are low (low systemic inflammation state). DII was not a moderator in the relationship between the protective association of physical activity and chronic pain.

The relationship between the protective effect observed in physical activity against chronic pain can be explained by different mechanisms [26]. Evidence suggests that the improvement in pain outcomes related to reduction in body fat and also, as a consequence, reduction in BMI is associated with a significant decrease in overload weight on joints, especially in knees, hips and the spine, improvement in joint alignment, and also, improvement in posture [27].

Another well-studied pain inhibitory mechanism is physical activity-induced hypoalgesia, characterized by decreased pain sensitivity and perception to noxious stimulation immediately after an acute exercise [28,29]. The release of endogenous opioids has been hypothesized as a primary mechanism responsible for exercise-induced hypoalgesia. Physical activity activates the peripheral and central opioidergic system, and the concentration of beta-endorphin in plasma is five times higher after physical activity (e.g., running), and the activity in pain-modulatory opioid-related areas in the brain, such as periaqueductal gray is significantly altered [30].

A previous study using a conditioned pain modulation test showed that a reduced pain inhibitory capacity is present in different chronic pain conditions [31]. Although, self-reported levels of vigorous physical activity were also shown to be related to better functioning of endogenous pain modulatory systems, being associated with more efficient condition pain modulation in healthy adults [32]. Also, sustained sedentary behavior has a negative relationship with brain responses in areas involved in pain modulation, where a sedentary lifestyle could have detrimental effects on brain function integral to the descending inhibition of pain [33]. Physical activity acts by reducing the levels of those inflammatory proteins and guiding the immune system response by favoring an anti-inflammatory status [34]. Also, some studies suggested that physical activity can induce hypoalgesia by peripherical modulation of the transduction, transmission and processing of noxious stimuli [35], and through the systemic activation of the endogenous opioid, serotoninergic system and the central activation of the cortico-thalamic descending inhibitory pathways [36].

On the other hand, interestingly for joint pain, during physical activity intrinsic factors such as mechanical alignments, body weight, and muscle strength, and extrinsic factors like external forces and overload on the joint might play a crucial role as a risk factor for pain. Based on this, vigorous activity could result in an extrinsic overload factor acting against some intrinsic factors, such as mechanical misalignment and muscle weakness, promoting a risk for chronic pain instead of a protective factor. According to a study by Slemenda and colleagues [37], individuals with radiographic signs of pain because of ostearthritis exhibited an average 20% weaker quadriceps muscle compared to those without, even after adjusting for body mass and other relevant factors. This weakness was observed before the onset of pain, indicating that insufficient quadriceps strength could predispose individuals to developing symptoms later on. One implication of quadriceps weakness is reduced knee stability during activities that pose a risk, whether occupational or recreational activity.

**Figure 2:**
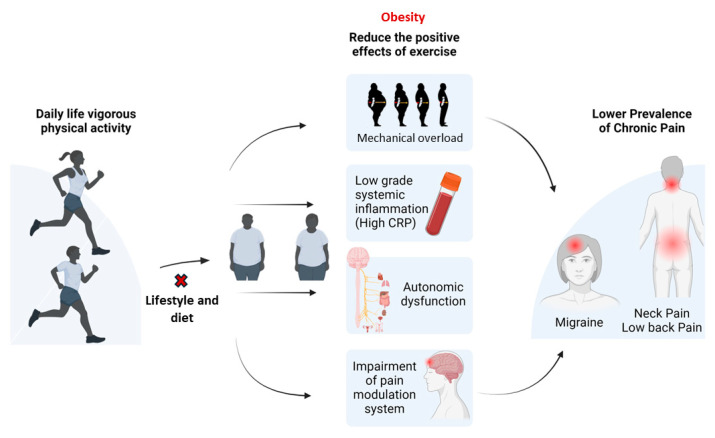
Theoretical mechanisms of obesity in physical activity for chronic pain.

Moreover, in this study, we found that vigorous physical activity has a protective effect against chronic pain in non-obese individuals, but this effect is not found in obese populations, suggesting that obesity alters the compensatory mechanisms associated with physical activity in chronic pain patients (Figure 2). Several mechanisms could explain this moderation effect. Obesity is related to adiposopathy, a state of excessive body weight, and dysfunctional fat tissue as a consequence of the enlargement of fat cells, which leads to elevated concentration of pro-inflammatory cytokines and biomarkers of inflammation (e.g., prostaglandins, C reactive protein) in the systemic circulation, secreted by adipose tissue. Adiposopathy leads to chronic systemic inflammation, which contributes to chronic pain, due to the activation of sensitized nociceptors lowering their thresholds of activation and amplifying the nociceptive input to the central nervous system [38].

Another possible explanation is the negative effects of obesity in the autonomic system and endocannabinoid/serotoninergic signaling [39,40,41,42]. Physical activity stimulates the endocannabinoid system, eliciting analgesic effects, reducing anxiety and depression, enhancing cognition, and producing various other effects. Physical activity augments serotonin levels in the brain, crucial for emotional processing and memory functions in the hippocampus, and also induces a dopamine surge mediated by the endocannabinoid system from the nucleus accumbens. A growing body of evidence indicates the interconnectedness of physical activity, the endocannabinoid system, and neurological health. Several studies have demonstrated associations between the endocannabinoid system and inflammatory diseases. Metabolic disorders such as obesity appear to be correlated with elevated circulating levels of endocannabinoids. Endocannabinoid receptor activity appears to be heightened in obesity, leading to increased lipogenesis, elevated food intake, and enhanced fat storage, leading to insulin resistance, type 2 diabetes, dyslipidemia and other inflammatory conditions, contributing to chronic pain [43,44,45]. Endocannabinoids also exhibit affinity for the vanilloid receptor (transient receptor potential vanilloid, TRPV), initiating peripheral and central nervous system signaling cascades. Upon binding, the ligand-receptor complex modulates nociception within the central nervous system. Consequently, endocannabinoids and their receptors play a role in activating nociceptors, thus contributing to pain sensation. Endocannabinoids derived from arachidonic acid serve as ligands for PPARA and PPARG (nuclear peroxisome proliferator-activated receptors), engaging in the regulation of carbohydrate and lipid metabolism, thereby supporting inflammatory responses [46]. These modifications present in obese people could explain the attenuated association between physical activity and chronic pain.

In this study, no relation was found between DII and the protective effect of physical activity. Other studies have also failed to identify associations between the DII in other outcomes [47,48,49]. The lack of association might be partly due to the cross-sectional design and also because the dietary information is in regards to a single day of nutritional habits [50], and may not necessarily reflect the nutritional habits of the population studied. More reliable results would be obtained with an analysis of dietary patterns over a longer period using a food frequency questionnaire, which assesses the consumption of food groups considering quantity and frequency over a period of one month. The strengths of this study include (1) a large and representative sample size of US population; (2) an analysis of both waves from NHANES dataset, which made possible to compare and achieve consistent results across both waves analyzed; (3) a comparison of different outcomes for chronic pain; (4) access health- and lifestyle-related factors that allows adjustments for potentials confounders. One limitation of this study is that physical activity data were collected from NHANES and is self-reported, which renders this measure highly subjective. This bias was reduced by considering the metabolic equivalent (MET) scores for vigorous work-related physical activity and moderate work-related physical activity. Measures for mild physical activity were not provided, which limited the comparison of results for moderate and vigorous physical activity. Additionally, the interpretation of OR below 1 as indicative of a protective effect represents a methodological limitation, as this approach, although commonly used in observational research, does not establish causality and should be interpreted with caution.

To enhance clinical practice, our findings highlight the need for personalized physical activity recommendations that consider BMI, systemic inflammation, and chronic pain conditions. Current guidelines do not incorporate BMI as a moderating factor in physical activity prescriptions, yet our results suggest that obesity may attenuate the protective effects of physical activity against chronic pain. Healthcare professionals and physicians should take these nuances into account when advising patients, ensuring that individuals with obesity, joint pain, or inflammatory conditions receive tailored physical activity interventions. Rather than discouraging physical activity, clinicians should adapt physical activity recommendations to maximize benefits while minimizing potential risks. By integrating these insights into clinical practice, we can promote more effective, evidence-based strategies for managing chronic pain and improving patients’ outcomes.

## 5. Conclusions

This study confirms the protective effect of vigorous physical activity on chronic pain, particularly in the neck, lower back, and hip regions, as well as migraine. However, this association is strongly moderated by BMI, being evident only in non-obese individuals (BMI < 30 kg/m^2^). Additionally, the protective effect of vigorous physical activity against chronic migraine was observed only in individuals with low serum CRP levels, highlighting the influence of systemic inflammatory status. On the other hand, the DII was not identified as a moderator of this relationship. These findings were consistent across different NHANES cycles, reinforcing the importance of body composition and inflammation in the interaction between physical activity and chronic pain. The findings of our study may have clinical implications. Physical activity should be prescribed on an individualized basis for individuals with joint pain, and high BMI and systemic inflammation should be considered when implementing and personalizing community-based physical activity intervention for chronic pain conditions.

Future research should explore the mechanisms behind BMI and inflammation in moderating the protective effects of physical activity on chronic pain. Longitudinal research and objective measures of physical activity and biomarkers are needed to clarify causality. Additionally, investigating different physical activity modalities and the combined impact of dietary interventions could help refine personalized treatment strategies.

## Figures and Tables

**Table 1:** Characteristics of study participants, NHANES 2003–2004 and 2009–2010.

Character	2003–2004 n = 4703	2009–2010 n = 5106
**Age (years)**		
Mean (SD)	48.90 (18.5)	44.27 (14.1)
**Gender**		
Female	2433 (51.5%)	2632 (51.6%)
Male	2288 (48.5%)	2474 (48.5%)
**Race/Ethnicity**		
Mexican American	944 (20.0%)	1026 (20.1%)
Non-Hispanic Black	949 (20.1%)	963 (18.9%)
Non-Hispanic White	2468 (52.3%)	2245 (43.9%)
Other Hispanic	147 (3.1%)	576 (11.3%)
Other Race—Including Multi-Racial	213 (4.5%)	296 (5.8%)
**Country of birth**		
Born Elsewhere	469 (9.9%)	862 (16.9%)
Born in 50 US States or Washington, DC	3720 (78.8%)	3590 (70.3%)
Born in Mexico	531 (11.3%)	652 (12.8%)
**Educational level**		
9–11th Grade (Includes 12th with no diploma)	696 (14.7%)	802 (15.7%)
College Graduate or above	879 (18.6%)	1060 (20.8%)
High School Grad/GED or Equivalent	1182 (25.0%)	1174 (22.9%)
Less Than 9th Grade	656 (13.9%)	581 (11.4%)
Some College or AA degree	1298 (27.5%)	1477 (28.9%)
**Marital status**		
Divorced	435 (9.2%)	595 (11.7%)
Living with partner	294 (6.2%)	474 (9.3%)
Married	2561 (54.3%)	2605 (51.0%)
Never married	827 (17.5%)	1063 (20.8%)
Separated	127 (2.7%)	193 (3.8%)
Widowed	476 (10.1%)	172 (3.4%)
**Annual family income**		
$ 0 to $ 19,999	1.406 (30.2%)	1.167 (23.0%)
$20,000 to $24,999	1.474 (31.7%)	1.447 (28.5%)
$45,000 to $54,999	806 (17.3%)	847 (16.7%)
$75,000 and over	740 (15.9%)	439 (8.7%)
**Poverty income ratio**		
Mean (SD)	2.59 (1.6)	2.43 (1.7)
**Alcohol consumption (number of drinks/last 12 months)**		
Mean (SD)	5.24 (38.8)	5.07 (24.8)
**Smoking (number of cigarettes/day last 30 days**		
Mean (SD)	16.02 (44.3)	12.41 (9.9)
**CRP serum levels (mg/dL)**		
Mean (SD)	0.47 (0.9)	0.41 (0.8)
**Physical, mental and emotional limitations**		
No	3415 (97.3%)	3944 (98.4%)
Yes	95 (2.7%)	64 (1.6%)
**Sleep disorders**		
No	N.A.	4728 (92.6%)
Yes	N.A.	371 (7.3%)
**DII**		
Mean (SD)	−0.02 (1.5)	0.02 (1.6)
**DII (categorical)**		
0 (No—anti-inflammatory)	2028 (48.3%)	2269 (47.6%)
1 (Yes—pro-inflammatory)	2175 (51.8%)	2496 (52.4%)
**Physical activity**		
Vigorous	1243 (26.3%)	1019 (19.9%)
Moderate	2.464 (52.4%)	1.886 (36.9%)
**BMI (kg/m^2^)**		
Mean (SD)	28.36 (6.2)	29.29 (7.0)
Non-obese < 30 kg/m^2^	2.941 (67.2%)	3.034 (61.1%)
Obese ≥ 30 kg/m^2^	1.434 (32.8%)	1.928 (38.9%)
**Pain**		
Low back/hip/neck	1752 (37.3%)	1.508 (29.5%)
Migraine	939 (19.9%)	N.A.
Joint pain	2124 (45.2%)	N.A.

N.A.: Not Applicable.

**Table 2:** Protective effect of physical activity on neck, low back, and hip pain, NHANES 2003–2004 and 2009–2010.

		2003–2004			2009–2010		
		O.R.	C.I. (2.5–97.5%)	*p* Value	O.R.	C.I. (2.5–97.5%)	*p* Value
**Unadjusted**	Vigorous physical activity	0.820	0.704–0.955	0.014 ^a^	0.748	0.599–0.934	0.013 ^b^
	Moderate physical activity	0.755	0.603–0.945	0.012 ^a^	1.146	0.934–1.406	0.175
**Adjusted**	Vigorous physical activity	0.798	0.647–0.984	0.037 ^a^	0.629	0.474–0.833	0.004 ^b^
	Moderate physical activity	0.804	0.622–1.039	0.08	1.306	0.949–1.796	0.090

^a^ Adjusted for gender, age and overall dietary inflammatory index. Gender is a significant independent predictor. ^b^ Adjusted for gender, age, physical, mental, and emotional limitations, sleep disorders and BMI. Physical, mental, emotional limitations, and sleep disorders are significant independent predictors.

**Table 3:** Protective effect of physical activity on migraine and joint pain, NHANES 2003–2004.

			O.R.	C.I. (2.5–97.5%)	*p* Value
**Unadjusted**	Joint pain	Vigorous physical activity	0.860	0.726–1.018	0.077
		Moderate physical activity	0.925	0.783–1.092	0.331
	Migraine	Vigorous physical activity	0.781	0.640–0.952	0.018 *
		Moderate physical activity	0.904	0.731–1.116	0.324
**Adjusted**	Joint pain	Vigorous physical activity	1.366	1.031–1.810	0.033 *
		Moderate physical activity	1.144	0.866–1.509	0.296
	Migraine	Vigorous physical activity	0.697	0.517–0.939	0.022 *
		Moderate physical activity	0.848	0.665–1.081	0.162

*: results where *p* < 0.05.

**Table 4:** Effect modification by BMI in neck, low back, and hip pain.

		2003–2004			2009–2010		
		Interaction *p* Value = 0.015 *			Interaction *p* Value = 0.011 *		
		O.R.	C.I. (2.5–97.5%)	*p* Value	O.R.	C.I. (2.5–97.5%)	*p* Value
**BMI**	Obese ≥30 kg/m^2^	0.924	0.741–1.152	0.449	0.75	0.530–1.062	0.096
	Non-obese <30 kg/m^2^	0.637	0.460–0.883	0.011 ^a^	0.568	0.429–0.752	0.0011 ^b^

^a^ Adjusted for age, gender, and dietary inflammatory index. Gender and dietary inflammatory index are significant independent predictors. ^b^ Adjusted for gender, age, physical, mental, and emotional limitations and sleep disorders. Gender and sleep disorders are significant independent predictors. *: results where *p* < 0.05.

**Table 5:** Effect modification by CRP in migraine.

		2003–2004		
			Interaction *p* Value = 0.006 **	
		O.R.	C.I. (2.5–97.5%)	*p* Value
**CRP**	High CRP (≥0.22 mg/dL)	1.198	0.783–1.831	0.364
	Low CRP (<0.22 mg/dL)	0.560	0.339–0.924	0.027 *

* Adjusted for age, gender, physical, mental and emotional limitations and BMI. ** results where *p* < 0.05.

## Data Availability

Data are contained within the article and the NHANES data files are available on the NHANES website.
